# Types and Characteristics of Fatal Accidents Caused by Multiple Processes in a Workplace: Based on Actual Cases in South Korea

**DOI:** 10.3390/ijerph19042047

**Published:** 2022-02-11

**Authors:** Sung-Yong Kang, Seongi Min, Won-Seok Kim, Jeong-Hun Won, Young-Jong Kang, Seungjun Kim

**Affiliations:** 1 School of Civil, Environmental and Architectural Engineering, Korea University, Seoul 02841, Korea; jokeksy@korea.ac.kr (S.-Y.K.); tjsrl541@korea.ac.kr (S.M.); yjkang@korea.ac.kr (Y.-J.K.); 2 Occupational Safety Research Department, Occupational Safety and Health Research Institute, Ulsan 44429, Korea; wonsok96@kosha.or.kr; 3 Department of Safety Engineering, Chungbuk National University, Cheongju 28644, Korea; jhwon@chungbuk.ac.kr

**Keywords:** industrial fatal accidents, multiple processes in the workplace, actual fatal accident cases, occupational safety and health policy

## Abstract

As the social cost of disasters increases and safety is being emphasized, policy regulations at the national level have been implemented. However, various fatal accidents are continually occurring as continued economic development and enhanced technologies have increased demand and complicated the industrial structure. Workers in different industries, performing similar jobs, often experience different workplace hazards, which can result in similar types of accidents. Therefore, new policy regulations have been established to separate multiple processes and work in workplaces and are being implemented in several countries to minimize damage caused by new types of industrial accidents. Supervision and management appropriate for contractors or safety and health officials with legal obligations are required to play a regulatory role when these types of industrial accidents are likely to occur. This study classified accidental types and their characteristics based on actual cases, in which potential risks exist at multiple processes in a workplace. First, raw data of work-related fatalities that occurred in South Korea were reviewed and classified as fatal accidents caused by multiple processes in workplaces using the proposed method. Next, the classified actual cases were prepared as statistical data and analyzed based on the various categories. Finally, the accident type based on multiple processes, including risks and characteristics, in workplaces was proposed. As a result, this study improved the safety awareness and understanding of regulatory subjects regarding industrial accidents caused by multiple processes in workplaces and is expected to improve the effectiveness of the existing policy to prevent workplace accidents.

## 1. Introduction

**Figure 1:**
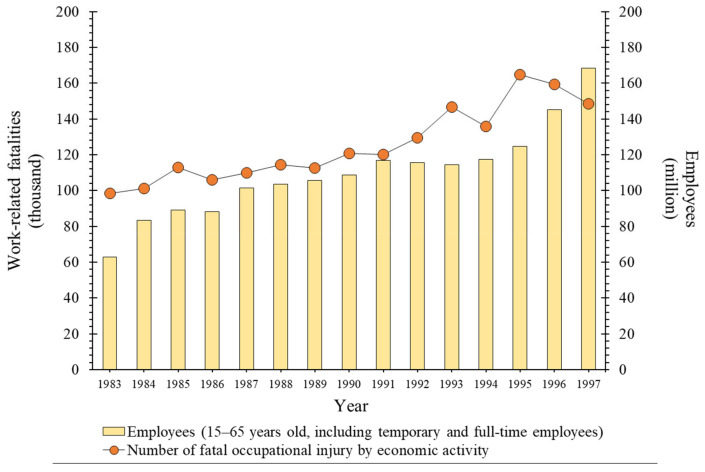
Number of employees and work-related fatalities worldwide at the end of the 19th century (data of 127 countries, including South Korea, Japan, Germany, and the United Kingdom, provided by the International Labour Organization (ILO)).

Since industrialization, humans have been using automated machines and heavy equipment to achieve greater production and increase production efficiency. To increase the rate and efficiency of production sufficiently met the demand of population growth and helped rapidly enhance industries in terms of scale and technology. As industries that met the domestic demand gradually expanded, communication and transportation developed, the global market expanded, and additional industries were established to meet demands of various countries. This happened in the last 100 years; several jobs have been created due to the increase in the global population and demand for industrial diversification, and this increase in the number of workers has also been associated with an increase in the number of fatal industrial accidents. In other words, owing to the development of industries, the number of workers steadily increased in terms of proportion, thereby increasing the number of industrial fatal accidents in workplaces until the end of the 20th century (Figure 1) [1]. This happens owing to an increase in the number of risky jobs, e.g., operation of various mechanical devices (manual and automatic), use of heavy equipment, and “work at height”.

As both the number and cost of fatal workplace accidents increased, there was a change in the perception of workplace safety, and policies to increase worker safety and prevent accidents were implemented at the national level [2]. In Japan, the “Occupational Accident Prevention Plan” was implemented to protect the safety and life of workers in industries. In Germany, the improvement of industrial technology and work environment has been promoted through “Industrie 4.0” and “Arbeiten 4.0”. In the UK, one of the occupational safety and health executives has created and implemented an annual “Health and Safety Executive Business Plan” to ensure the safety and health of employees [3].

As a result of the expansion of the global market after the economic crisis in 2008, which sharply increased demand and resulted in a recovery of the economy, it was necessary to optimize supply and establish mass-production systems. In addition to these demands, owing to technological developments and changes (construction and maintenance according to aging facilities, production of alternative energy to respond to climate change, and export and import of large-scale materials) in technology, the current industry is complex with various processes and simultaneous production activities. A new type of industrial accident is emerging between workers who simultaneously conduct the different works at the same workplace at the same time. When several workers with different affiliations (e.g., contractor–subcontractor or subcontractor–subcontractor) are performing different tasks in the same workplace, interfering with each other’s work without the entire work process notice and the proper safety coordination, it can lead to large-scale industrial accidents. To prevent this, government-level regulations are being implemented in some countries. In Japan, “Articles 15 and 30 of the Occupational Safety and Health Act” state that the general safety and sanitation manager or a specific prime contractor can prevent accidents caused by work performed at the same place [4,5,6]. In Germany, “Article 8 of the Occupational Safety and Health Act” stipulates that multiple subcontractors should comply with safety and health regulations when workers operate in the same workplace as the subcontractors. In addition, “Chapter 2 of the Accident Prevention Regulations of the Legal Accident Insurance Corporation” states that a person in charge of safety coordination must be designated to prevent risks occurring in the same place [7,8,9]. In the UK, in the construction industry, the law requires a structural designer or contractor to coordinate safety issues during the construction phase that includes multiple simultaneous or sequential operations [10]. In these countries, institutional mechanisms are in place to protect workers when multiple processes are simultaneously performed in a workplace (henceforth, multiple processes in the workplace); however, legal obligations are imposed by designating a person who would be in charge within the approximate range of the risk of an accident. However, for managers to implement effective safety and health checks and measures with legal obligations, it is necessary to clarify the scope based on types of accidents that are likely to occur in actual industrial sites and to specify the characteristics of each accident.

**Figure 2:**
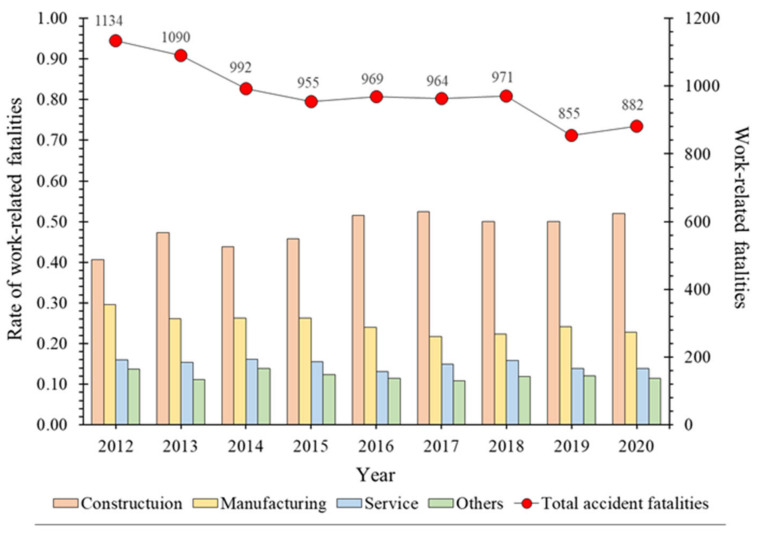
Annual number of fatalities and rate of fatalities in the industry.

With regard to the status of industrial accidents in South Korea (henceforth, Korea), there has been a steady decrease in work-related fatalities since 2012 (Figure 2) [11]. This implies that the “5-year Industrial Accident Prevention Plan”, implemented to achieve the industrial accident prevention goal within a certain period was effective [12]. On the other hand, in the case of the construction industry, the ratio of industrial accidents is increasing unlike other industries, and most accidents are caused by falls. This hazard is due to the use of improper temporary structures as well as the unsafe behavior of workers [13,14,15,16,17]. Therefore, to prevent industrial accidents in the construction industry, the Korea Occupational Safety and Health Agency (KOSHA), an organization under the Ministry of Employment and Labor (MOEL), is implementing policies such as “Evaluation of Construction Company Industrial Accident Prevention Activities”, “Hazard and Risk Prevention Plan Review”, and “KOSHA-Management System” [18,19,20,21]. This is a policy to reduce fatal accidents in the construction industry.

Despite these efforts, large-scale industrial accidents have continuously occurred due to the multiple processes in the workplace. In 2014, a fire started by sparks from gas pipe welding work spread to the ceiling and nearby combustible materials in the interior design workplace, and it produced the toxic smoke that billowed upward. Due to the smoke, seven people died, and fifty people were injured [22]. Accordingly, based on the revision of the Occupational Safety and Health Act in 2017, in the construction industry, where there are multiple processes in the workplace, a system has been introduced that allows the main party to designate a safety and health coordinator for inspection and adjustment [23,24,25]. Moreover, accidents caused by multiple processes in the workplace occurred in other industries. In 2017, a fatal accident (six deaths, twenty-five injuries) occurred owing to a collision caused by the interference of the lift radius of a crane during manufacturing [26]. Owing to this repetitive occurrence in all the industries, in 2021, the Occupational Safety and Health Act has been revised to prevent accidents by multiple processes in the workplace (Table 1) [27]. In addition, the act states that contractors should confirm and coordinate safety and health measures for work (contractors and subcontractors in the same workplace).

In other countries, a method is required to improve the effectiveness of accident prevention policies as regulations to prevent accidents caused by multiple processes in the workplace do not provide accurate information on accident types and specific characteristics. This study developed a procedure to determine types of accidents caused by multiple processes in the workplace based on actual data of fatal accidents that occurred in major industrial sites in Korea. In addition, statistical analysis was performed by converting fatal accident cases (caused by multiple processes in the workplace), which were investigated, into numerical data. Finally, by proposing risks and characteristics of each accident type based on actual cases, it is intended to improve safety awareness and the understanding of accidents caused by multiple processes in the workplace of contractors. Furthermore, if the proposed method and risks and characteristics of each type of accident mentioned in this study are used in actual case-based studies in other countries, an improvement is expected with regard to the effectiveness of the accident prevention policy due to multiple processes in the workplace.

## 2. Determination Procedure and Actual Cases of Fatal Accidents Caused by Multiple Processes in the Workplace

### 2.1. Procedures and Decision Criteria to Determine Fatal Accidents Caused by Multiple Processes in the Workplace

To determine fatal accidents caused by multiple processes in the workplace, 4641 raw cases were analyzed over the past 5 years (2016–2020) using “statistical data on related-work fatal accidents” (provided by the Ministry of Employment and Labor) and “fatal accident investigation opinions” (provided by Korea Occupational Safety and Health Agency). In particular, the fatal accident investigation opinion provides a variety of information, e.g., exact accident summary, source, affiliation of worker, process, type of work, and stakeholder relationship. As these reports contain personal information about victims, filtered data was provided from the institute.

A decision criterion is needed to determine whether an accident occurred due to multiple processes in the workplace. Therefore, based on the actual data, the literature on the accident analysis model that analyzes the causal relationship to an accident was investigated. First the ConAC (Construction Accident Causation) framework analyzes workplace accidents of varying levels of severity, however does not take into account process interference in time and space as it focuses on determining the causes of accidents within the same process [28,29]. Next, in the case of arranging different accident types according to the frequency in Bow-Tie form using Stroybuilder, it is possible to find a common accident path for each case, however it may be difficult to generalize the path in multiple processes [30,31]. For this reason, in order to construct the causal relationship of accidents due to multiple processes in the workplace, the common criteria stakeholders, process, time, and space were established as shown in Table 2 for the decision procedure in actual cases.

Cases where multiple processes in the workplace were executed were selected as stakeholder relationships, process interference, and space–time interference, and each case was classified based on notations.

**Figure 3:**
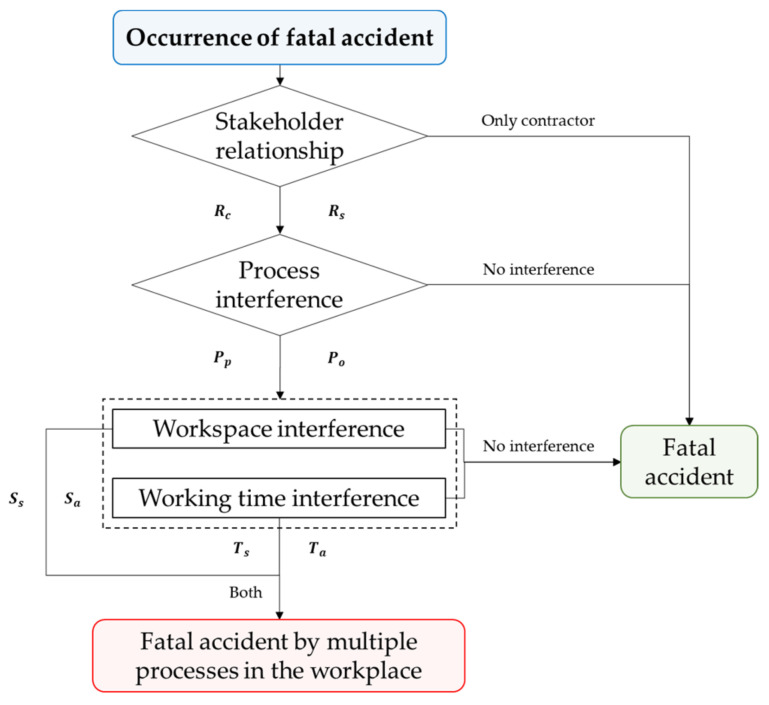
Procedures to determine accidents caused by multiple processes in the workplace.

Figure 3 shows the procedure to determine whether a fatal accident that occurred owing to the developed standard was an accident caused by multiple processes in the workplace.

The relationship between the stakeholders in the accident that occurred in Step A was identified; it was determined that an accident caused by multiple processes in the workplace could occur while working under a contractor and subcontractor (or subcontractors). On the other hand, if only a contractor was involved, it was classified as an accident rather than an accident caused by multiple processes in the workplace as it was not subjected to the regulation outlined in the “Occupational Safety and Health Act Article 64”.

In Step B, we determined whether there was any interference in the process or operation, whether two or more processes were in progress, and whether there could be interference when performing other tasks (maintenance, inspection, confirmation, cleaning, removal, and inspection) when machines were operating.

In Step C, we determined whether there was any interference between the workspace and time. If space and time are separated, accidents caused by multiple processes in the workplace do not occur; therefore, if one of these dimensions does not interfere, it is classified as an accident rather than an accident caused by multiple processes in the workplace. This characteristic can be used in future studies on accident prevention measures with regard to multiple processes in the workplace. Therefore, when the aforementioned procedures were satisfied, it was determined as an accident caused by multiple processes in the workplace.

### 2.2. Analysis of Representative Cases of Industries According to the Developed Determination Procedure

According to the developed determination procedure, representative cases of fatal accidents caused by multiple processes in the workplace in construction, manufacturing, service, and other industries were investigated. The procedures and content of the representative cases were prepared based on the investigation of fatal accidents provided by the Korea Occupational Safety and Health Agency.

#### 2.2.1. Construction Industry

In the case of the construction industry, there is a high risk of multiple processes being performed simultaneously in the same workplace by contractors and/or subcontractors. In addition, heavy equipment is operated, heavy objects are transported, and temporary facilities are used, potentially increasing the risk of fatal accidents, which have been observed in several investigated cases. Table 3 lists the representative actual cases of accidents caused by multiple processes in the workplace during construction.

#### 2.2.2. Manufacturing

Manufacturing workplaces include shipbuilding, steelmaking, chemical industries and plants, and product factories. Most of them use manual or automatic rotating machinery and cranes to transport heavy loads. Therefore, there is a potential risk of exposure to chemicals, electric shock, and suffocation, as well as physical hazards. In actual cases, accidents caused by multiple processes in the workplace were investigated (Table 4).

#### 2.2.3. Service and Other Industries

Although the number of accidents caused by multiple processes in the workplace in service and other industries is smaller than that in the construction or manufacturing industry, accidents are caused by multiple processes in various workplaces. Industries classified as services include logistics, telecommunications, finance, and energy supply. Others include primary industries such as agriculture, mining, forestry, fishing, and so on. Table 5 lists actual cases of accidents caused by multiple processes in the workplace in the service and other industries.

## 3. Classification of Accidental Types and Their Characteristics Based on Statistical Analysis of Actual Fatal Accidents Caused by Multiple Processes in the Workplace

As shown in Figure 2, Korea has introduced various industrial accident prevention measures and legal regulations, and fatalities owing to occupational accidents are gradually decreasing. However, new types of accidents occur continuously owing to multiple processes in the workplace, and fatal accidents are being reported. In 2016, a worker of a subcontractor got caught in a door while repairing a subway screen door and died; since then, there has been an increase in the attention paid to accidents caused by multiple processes in the workplace. This fatal accident started to raise a new issue of “outsourcing of risk” and the blind spot for safety and health management supervision for disasters caused by the contract. Accordingly, the government revised the Occupational Safety and Health Act to expand the scope to identify and consider measures for places managed by contractors from the existing 22 to all places. While the revision was in progress, another accident took place due to the implementation of multiple processes in the workplace. In 2017, an accident occurred when the working radius of the main and auxiliary cranes collided in the shipyard. In 2018, an employee of a subcontractor died during the inspection of a power plant transportation facility. These accidents occurred owing to insufficient safety and health supervision/action/coordination in the relationship between contract and supply, and thus, the government introduced new preventive measures. Therefore, in “Occupational Safety and Health Act Article 64 No. 7, No. 8”, the contractor’s responsibility to prevent multiple processes in the workplace has been strengthened. This is a policy, in which a contractor prevents accidents caused by multiple processes in the workplace, when the process or work performed in the workplace interferes with other processes of a subcontractor or between different subcontractors. For the effective implementation of these policies, it is necessary to improve the awareness and understanding of accidents caused by multiple processes in the workplace among contractors subject to regulation. Therefore, to improve the effectiveness of policies, information on risks and characteristics of each type of accident were derived based on accidents caused by multiple processes in the workplace.

### 3.1. Statistical Analysis of Fatal Accidents Caused by Multiple Processes in the Workplace

Statistical data on work-related fatal accidents provided by the Ministry of Employment and Labor does not indicate the proportion of accidents caused by multiple processes in the workplace. Therefore, the raw data of fatal accident investigation opinions were analyzed based on the proposed decision procedure to classify the accidents by multiple processes in the workplace. The analysis results were quantified to determine the proportion of the number of fatalities caused by multiple processes in the workplace. The statistics of fatal accidents caused by multiple processes in the workplace were analyzed based on industry, accident type, and source.

**Figure 4:**
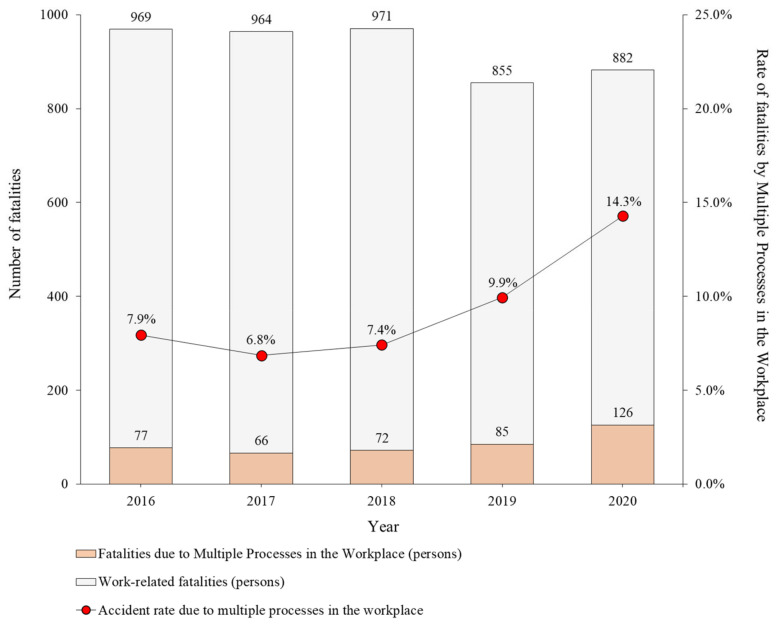
Percentage of fatalities caused by multiple processes in the workplace among work-related fatalities (2016–2020).

Figure 4 compares the number of deaths caused by multiple processes in the workplace. Over the past 5 years, 4641 work-related fatalities occurred, of which 426 (9.2%) were fatal accidents caused by multiple processes in the workplace. If the ratio is expressed by year, fatality due to multiple processes in the workplace sharply increased after 2017 (66 people), reaching a maximum of 14.3% (126 people) in 2020.

**Figure 5:**
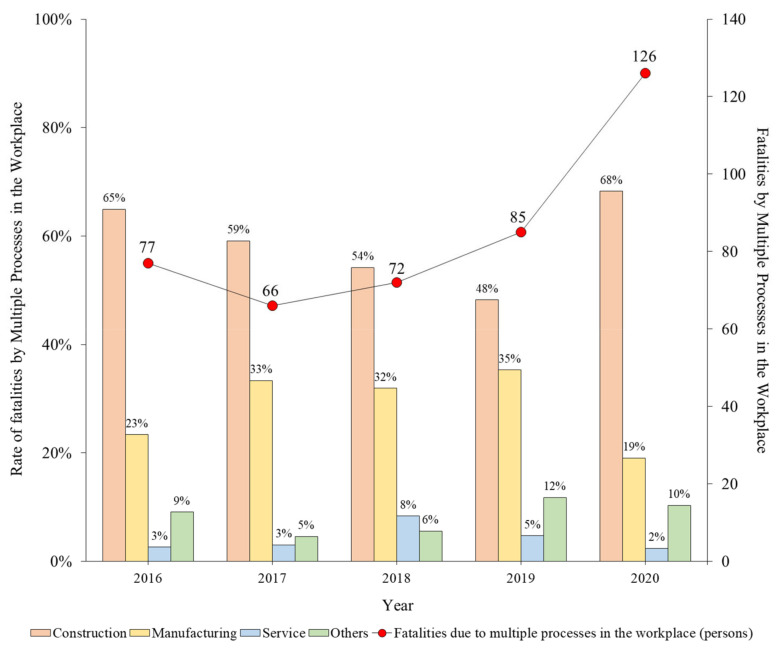
Percentage of fatalities caused by multiple processes in the workplace based on industry (2016–2020).

Table 6 classifies fatalities caused by multiple processes in the workplace based on industry (Figure 5). The distribution of fatalities based on industry is similar to the distribution in Figure 2, in the order of construction, manufacturing, services, and others. In the case of construction, after 2017, there is a decreasing tendency, which indicates that the safety and health coordinator system implemented in 2017 effectively contributed to the prevention of accidents caused by multiple processes in the workplace. However, in 2020, the proportion of fatalities caused by multiple processes in the workplace significantly increased.

**Figure 6:**
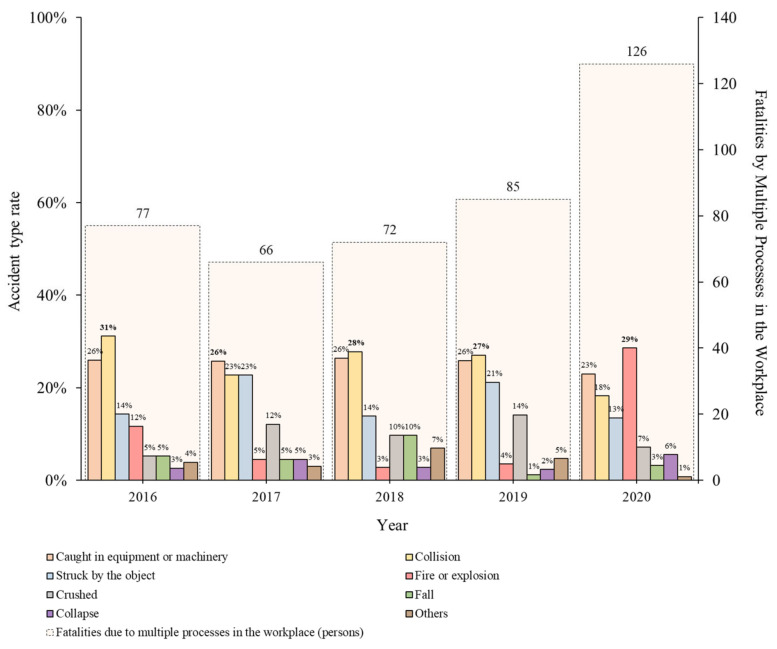
Percentage of types of fatalities caused by multiple processes in the workplace (2016–2020).

Table 7 lists the ratio of accidents caused by multiple processes in the workplace based on the accident type (Figure 6). In the actual case, accidents caused by multiple processes in the workplace can be classified into eight categories: “caught in equipment or machinery”, “collision”, “struck by objects”, “fire or explosion”, “crushed”, “fall”, “collapse”, and others. The accident types, “Caught in equipment or machinery”, “struck by objects”, and “collision”, were analyzed to maintain high frequency every year. In 2020, a large-scale fire accident occurred owing to the execution of multiple processes in the workplace during construction, and “fire and explosion” exhibited the highest rate. This is related to high fatalities caused by multiple processes in the workplace in the construction industry in 2020, which shows the status of each industry (Figure 5). As a result of analyzing fatal accident investigation opinions, it was confirmed that there was one fire accident caused by multiple processes in the workplace during construction, and 36 deaths were recorded. In the construction industry, most accidents are caused by falls and collisions, however accidents caused by multiple processes in the workplace show a completely different aspect. Therefore, in the case of accidents caused by multiple processes in the workplace in all the industries, including the construction industry, a wide range of risks is inherent. To identify and remediate incidents caused by multiple processes in the workplace, contractors need to understand types of accidents that can occur in industries.

**Figure 7:**
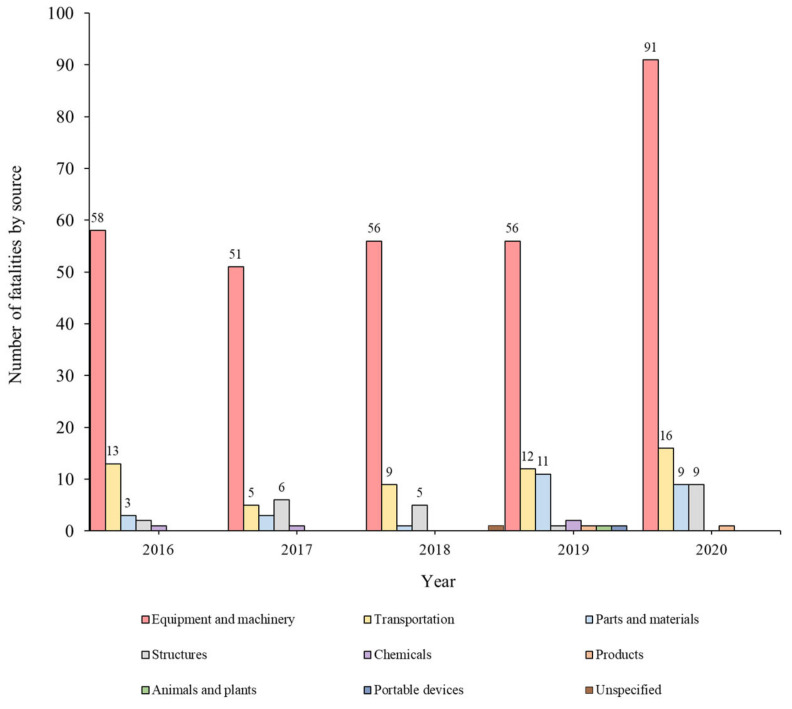
Sources of accidents caused by multiple processes in the workplace.

**Figure 8:**
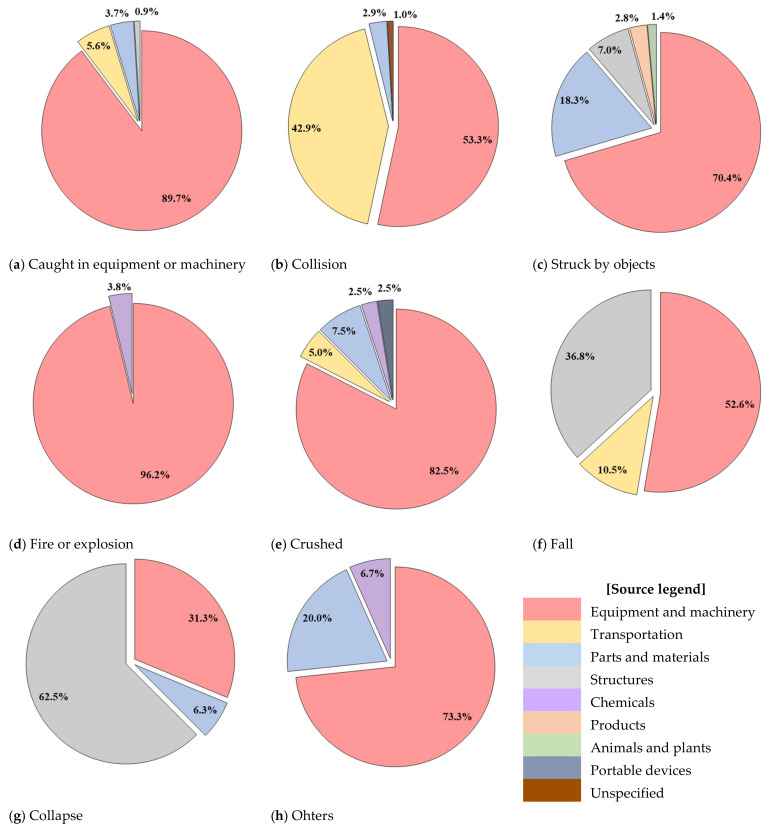
Distribution of sources of accidents caused by multiple processes in the workplace.

Figure 7 and Figure 8 show the distribution of sources of accidents caused by multiple processes in the workplace and the ratio based on the accident type, respectively, and the detailed causes were investigated based on the fatal accident investigation opinions.

The most common source, “Equipment and machinery”, accounts for a very high proportion of causes of all accident types every year. In particular, “Fire or explosion (96.2%)”, “Caught in equipment or machinery (89.7%)”, and “Crushed (82.5%)” exhibited very high rates. Among them, in the case of “Fire or explosion”, it was found that sparks generated by the operation of mechanical devices for welding or cutting caused fire.

“Transportation” was responsible for “Collision (42.9%)”, “Fall (10.5%)”, “Caught in equipment or machinery (5.6%)”, and “Crushed (5.0%)”. Most accidents had occurred owing to transportation related to the transport of heavy objects or construction materials in the workplace.

The “Parts and materials” source was responsible for “Struck by objects (18.3%)”; it occurred when “Parts or materials” fell from a high place or partly detached from a mechanical device.

The “Structures” source was responsible for “Collapse (62.5%)” and “Fall (36.8%)”; the source was provided when the structure was overturned by an external force or the whole structure collapsed.

In addition, “Chemicals”, “Products”, “Animals and plants”, and “Portable devices” were the source of accidents caused by multiple processes in the workplace, although the frequency was found to be very low.

Table 8 shows the fatalities and ratio of fatalities by accident type and source due to multiple processes in the workplace by industry.

In the case of construction, all the accidents were caused by “Equipment and machinery”; “Fire and explosion” caused the highest number of accidents. In addition, “Collisions” caused by “Transportation”, “Struck by objects” caused by “Parts and materials”, and “Collapse” caused by “Structures” were investigated.

In manufacturing, “Caught in equipment or machinery” caused the highest number of accidents. Moreover, accident types were investigated based on the sources.

In the case of the service and other industries, accidents were caused by various sources; however, the rate of fatalities was low owing to the low proportion.

### 3.2. Classification of Accidental Types Caused by Multiple Processes in the Workplace

To derive accident types, statistical data were prepared by determining fatal accidents (work-related) caused by multiple processes in the workplace during 2016–2020 in Korea. Based on the fatal accident investigation opinions, the accident occurrence process and source, process, time, and space interference were investigated, and eight representative accident types were found. As a result, the risk that could cause an accident owing to the execution of multiple processes in the workplace was defined as the interference between the process and work, and characteristics were classified as the interference of time and space. Therefore, according to each accident type, risks and characteristics that can cause death owing to the execution of multiple processes in the workplace were explained (Table 9).

## 4. Conclusions

As fatal accidents caused by multiple processes in the workplace became an issue throughout industries, Korea enacted “Occupational Safety and Health Act Article 64” to strengthen the obligations of contractors. Several countries already have policies related to reducing accidents by multiple processes in the workplace, however, they are limited to the construction and manufacturing industries. On the other hand, in Korea, the scope of these laws is being extended to all industries. Accordingly, the types of accidents that occur due to multiple processes in the workplace, and their characteristics, should be clearly defined and investigated for proper operation and classification of responsibility, without any legal confusion. Therefore, actual cases of work-related fatalities that occurred in major industries in Korea during 2016–2020 were analyzed, and accident cases caused by multiple processes in the workplace were identified using the proposed objective procedure. Deaths caused by multiple processes in the workplace were statistically analyzed based on industry, accident type, and source. As a result, it was confirmed that fatalities gradually increased owing to multiple processes in the workplace in the industry.

In this study, the following points were concluded by performing a detailed analysis based on statistical data and actual cases.

- Based on the criteria and procedures to classify multiple processes in the workplace, 4641 cases of occupational accidents and deaths in Korea over the past 5 years were analyzed and quantified using statistical data. According to the statistical data, 426 (average 9.2%) of the occupational accidents that occurred over the past 5 years were considered fatal accidents caused by multiple processes in the workplace. Moreover, from 2017 (66 persons, 6.8%) to 2020 (126 persons, 14.3%), the graph exhibited a tendency to increase every year. Among all the industries, the distribution of deaths caused by multiple processes in the workplace was found to be more than 87% in the construction (average 58.9%) and manufacturing (average 28.6%) industries. In addition, it was 4.2% (on average) in the service industry and 8.3% (on average) in other industries. In addition, it was 4.2% (on average) in the service industry and 8.3% (on average) in other industries.
- The accident types were reviewed based on statistical data and fatal accident investigation opinions. Accidents caused by the “Caught in equipment or machinery” and “Collision” types occurred at a high rate every year, and fatalities were the highest. On the other hand, accidents where the cause was listed as “Struck by the object”, “Fire or explosion”, and “Crushed” occurred less frequently; however, a large number of fatalities occurred in each case. Most accidents with regard to “Fall” were caused by an external impact or object interference from the work platform. “Collapse” is less likely to cause accidents with regard to the execution of multiple processes in the workplace, and it has been investigated whether those accidents occurred due to non-compliance with the process sequence and insufficient safety control and preparation with regard to the work assigned by a contractor.
- Sources were analyzed as they had a direct relationship with causes of accidents owing to the execution of multiple processes in the workplace. Among the sources of accidents caused by multiple processes in the workplace, the highest fatalities were caused by “equipment and machinery”. Moreover, this was confirmed in the relationship with the accident type, and most accidents are related to interference with the operation of equipment and machines near workers. With regard to the source based on “Transportation”, accidents related to “Collision” were maximum in numbers, and with regard to source based on “Parts and materials”, accidents related to “Struck by objects” were maximum in numbers. “Structure” was the main source of “Collapse”, and the remaining causes exhibited a low frequency of occurrences.
- The following eight accident types were derived based on actual cases of fatal accidents caused by multiple processes in the workplace: “Caught in equipment or machinery”; “Collision”; “Struck by objects”; “Fire or explosion”; “Crushed”; “Fall”; “Collapse”; and others. This category is based on fatal accident investigation opinions, and work-related fatalities provide statistical data and actual cases. Therefore, the source and occurrence investigated in the actual case were analyzed in detail, and the risks and characteristics of the accident types that can cause death owing to the execution of multiple processes in the workplace were explained based on the interference of work and process, space, and time.

In this study, accidents caused by multiple processes in the workplace were separated from work-related fatalities, and the tendency and details of fatal accidents caused by multiple processes in the workplace were analyzed. Based on the analyzed results, the risks and characteristics of accident types that can cause fatal accidents owing to multiple processes in the workplace were proposed. In other words, to increase the awareness and understanding of the risk of accidents caused by multiple processes in the workplace, as defined by the law, the risk and characteristics according to the accident type were suggested based on cases. It is expected to inform workers and contractors about the possibility and risk of accidents caused by mixed work inherent throughout the industry.

Although this study collected accident cases according to industrial classification in Korea, a common criterion is proposed so that it can be applied to all industries in the process of determining accidents due to multiple processes in the workplace. To recognize the importance of, and prevent, such disasters in other countries, it is necessary to be able to classify disasters caused by multiple processes in the workplace specifically. Through the procedure and case-based study data to distinguish the types and characteristics proposed in this study, it is expected that the risk of accidents caused by multiple processes in the workplace in countries around the world, and the need for prevention, will be recognized. Based on analyses of workplace accidents due to multiple procedures, policies and manuals for preventing these types of accidents can be developed in other countries.

In further research, the mechanism of fatal industrial accidents due to the multiple processes in the workplace, as classified in this study, will be investigated based on real cases. Once the mechanism is established, the appropriate safety coordination, effective process management, and supervision methodologies can be suggested to minimize the probability of fatal accidents.

## Figures and Tables

**Table 1:** Occupational Safety and Health Act Article 64, Paragraph 1, No. 7, No. 8 (in Korea).

Article 64: Measures for Preventing Industrial Accidents in Contracting
Paragraph 1: A contractor shall consider the following measures when employees work under him or her: (Nos. 1–6 omitted) No. 7: In the work of a contractor/subcontractor in the same workplace, the work period and content of the contract, e.g., safety and health measures must be confirmed. No. 8: In addition to No. 7, if there is a risk, based on the presidential decree, of catching fire or explosion owing to the implementation of multiple processes in the workplace, the work period and content of the contract must be adjusted.

**Table 2:** Decision process criteria and notation of multiple processes in the workplace.

Step	Classification	Decision Criteria	Notation
A	Stakeholder relationship	■ Accidents occurred involving a contractor and a subcontractor	$R_{c}$
		■ Accidents occurred involving subcontractors	$R_{s}$
B	Process interference	■ Accidents caused by other processes	$P_{p}$
		■ Accidents caused by two or more operations within a process	$P_{o}$
C	Workspace interference	■ Accidents occurring in the same workplace	$S_{s}$
		■ Accidents occurring in adjacent workplaces	$S_{a}$
	Working time interference	■ Accidents occurring at the same working time	$T_{s}$
		■ Accidents caused by the previous process affecting continuous working time	$T_{a}$

**Table 3:** Representative actual cases of the accident caused by multiple processes in the workplace—Construction.

Case 1	Accident summary	During the urethane foam work on the first basement floor of the logistics center construction site, sparks occurred during welding and cutting of pipes on the first floor, resulting in a fire (36 dead, 12 injured).			
	Decision process	Step		Notation	Analysis
		A		$R_{s}$	Subcontractors
		B		$P_{p}$	Plumbing work and insulation work
		C	Workspace	$S_{a}$	Continuous piping work after not finishing the urethane foam work in the adjacent space
			Working time	$T_{a}$	
	Cause of disaster	Fire caused by sparks			
	Accident type	Fire or explosion			
	Source	Equipment and machinery			
	Classification	Multiple processes in the workplace			
Case 2	Accident summary	The victim was on a table lift to install a temporary wall inside the factory by welding. At the same time, the girder of the overhead fixed crane, which was transporting materials from an adjacent place, collided with the victim, causing a fall accident. (1 dead)			
	Decision process	Step		Notation	Analysis
		A		$R_{c}$	A contractor and a subcontractor
		B		$P_{p}$	Welding work and transport work
		C	Workspace	$S_{a}$	Temporary structure installation work and transport work carried out at the same time in an adjacent place
			Working time	$T_{s}$	
	Cause of disaster	Collision while moving equipment			
	Accident type	Collision			
	Source	Equipment and machinery			
	Classification	Multiple processes in the workplace			
Case 3	Accident summary	While lifting the workbench (portable climbing system) installed for curtain wall work on the outer wall of a skyscraper of height >200 m, the fixing bracket fell, and the entire structure fell to the floor. At the same time, workers who were pouring concrete from below collided with the structure. (4 dead)			
	Decision process	Step		Notation	Analysis
		A		$R_{c}$	A contractor and a subcontractor
		B		$P_{p}$	Curtain wall installation work and concrete pouring work
		C	Workspace	$S_{a}$	Upper exterior wall work and lower pour work at the same time in adjacent places
			Working time	$T_{s}$	
	Cause of disaster	Struck by a falling object			
	Accident type	Struck by objects			
	Source	Structures			
	Classification	Multiple processes in the workplace			

**Table 4:** Representative actual cases of accidents caused by multiple processes in the workplace—Manufacturing.

Case 1	Accident summary	In the shipyard an accident occurred, in which the jib and wire rope of the auxiliary crane fell due to collision caused by the interference of the turning radius during the lifting operation of the main and auxiliary cranes. At the same time, an accident occurred, in which workers working on the lower main deck collided with a falling object. (6 dead, 25 injured).			
	Decision process	Step		Notation	Analysis
		A		$R_{c}$	A contractor and a subcontractor
		B		$P_{p}$	Lifting cranes work and working on decks
		C	Workspace	$S_{a}$	A secondary accident occurred while working at the same time on the lower part during the crane lifting operation
			Working time	$T_{s}$	
	Cause of disaster	Struck by a falling object			
	Accident type	Struck by objects			
	Source	Parts and materials			
	Classification	Multiple processes in the workplace			
Case 2	Accident summary	A subcontractor worker got caught between the machine and floor while cleaning the floor of an automatic lift that transports food in a contractor’s factory. (1 dead)			
	Decision process	Step		Notation	Analysis
		A		$R_{c}$	A contractor and a subcontractor
		B		$P_{o}$	Production equipment operation and cleaning
		C	Workspace	$S_{s}$	Automated lift operation (power on) and cleaning at the same time
			Working time	$T_{s}$	
	Cause of disaster	Caught between rotating machines (both rotating in the opposite direction)			
	Accident type	Caught in equipment or machinery			
	Source	Equipment and machinery			
	Classification	Multiple processes in the workplace			
Case 3	Accident summary	A worker entered the pipe that was being welded to produce a gas carrier in the shipyard. The worker suffocated to death owing to lack of oxygen caused by argon gas while connecting the compressor pipe. (1 dead)			
	Decision process	Step		Notation	Analysis
		A		$R_{s}$	Subcontractors
		B		$P_{p}$	Plumbing work and welding work
		C	Workspace	$S_{s}$	Upper exterior wall work and lower pour work at the same time in adjacent places
			Working time	$T_{a}$	
	Cause of disaster	Depletion of oxygen			
	Accident type	Others (Suffocations)			
	Source	Chemicals			
	Classification	Multiple processes in the workplace			

**Table 5:** Representative actual cases of the accident caused by multiple processes in the workplace—Service and others.

Case 1	Accident summary	Subcontractor workers were hit by a running train while making repairs related to the power supply to the line. (3 deaths)			
	Decision process	Step		Notation	Analysis
		A		$R_{c}$	A contractor and a subcontractor
		B		$P_{p}$	Train operation and maintenance work
		C	Workspace	$S_{s}$	Perform maintenance at the same time on active train tracks
			Working time	$T_{s}$	
	Cause of disaster	Collision with the transport medium			
	Accident type	Collision			
	Source	Transportation			
	Classification	Multiple processes in the workplace			
Case 2	Accident summary	While moving the blasted rock from the quarry to the excavator, the rock broke off and hit a worker, who was dismantling the fence under the slope. (1 dead)			
	Decision process	Notation		Notation	Analysis
		$R_{c}$		$R_{c}$	A contractor and a subcontractor
		$P_{p}$		$P_{o}$	Production equipment operation and cleaning
		$S_{a}\;T_{s}$	Workspace	$S_{s}$	Automated lift operation (power on) and cleaning at the same time
			Working time	$T_{s}$	
	Cause of disaster	Struck by a falling object			
	Accident type	Struck by objects			
	Source	Parts and materials			
	Classification	Multiple processes in the workplace			
Case 3	Accident summary	The victim was installing structural H-beams in the cargo hold of a ship for logistics transport. At the same time, an accident occurred when a forklift that was carrying out the unloading work collided with the H-beam, and the victim was crushed. (1 dead)			
	Decision process	Step		Notation	Analysis
		A		$R_{s}$	Subcontractors
		B		$P_{p}$	Temporary installation work and shipping work
		C	Workspace	$S_{s}$	Shipment work is carried out at the same time in the same space as the temporary installation work
			Working time	$T_{s}$	
	Cause of disaster	Crushed by an overturned object			
	Accident type	Crushed			
	Source	Parts and materials			
	Classification	Multiple processes in the workplace			

**Table 6:** Fatalities caused by multiple processes in the workplace based on industry.

Year	2016		2017		2018		2019		2020	
Classification	Fatalities (Persons)	Ratio	Fatalities (Persons)	Ratio	Fatalities (Persons)	Ratio	Fatalities (Persons)	Ratio	Fatalities (Persons)	Ratio
Construction	50	64.9%	39	59.1%	39	54.2%	41	48.2%	86	68.3%
Manufacturing	18	23.4%	22	33.3%	23	31.9%	30	35.3%	24	19.0%
Service	2	2.6%	2	3.0%	6	8.3%	4	4.7%	3	2.4%
Others	7	9.1%	3	4.6%	4	5.6%	10	11.8%	13	10.3%
Total	77		66		72		85		126	

**Table 7:** Fatalities and ratio based on the accident type caused by multiple processes in the workplace.

Year	2016		2017		2018		2019		2020	
Classification	Fatalities (Persons)	Ratio	Fatalities (Persons)	Ratio	Fatalities (Persons)	Ratio	Fatalities (Persons)	Ratio	Fatalities (Persons)	Ratio
Caught in equipment or machinery	20	26.0%	17	25.8%	19	26.4%	22	25.9%	29	23.0%
Collision	24	31.1%	15	22.8%	20	27.8%	23	27.0%	23	18.2%
Struck by the object	11	14.3%	15	22.8%	10	13.9%	18	21.2%	17	13.5%
Fire or explosion	9	11.7%	3	4.5%	2	2.8%	3	3.5%	36	28.6%
Crushed	4	5.2%	8	12.1%	7	9.7%	12	14.1%	9	7.1%
Fall	4	5.2%	3	4.5%	7	9.7%	1	1.2%	4	3.2%
Collapse	2	2.6%	3	4.5%	2	2.8%	2	2.4%	7	5.6%
Others	3	3.9%	2	3.0%	5	6.9%	4	4.7%	1	0.8%
Total	77		66		72		85		126	

**Table 8:** Fatalities and ratio by sources of accident types due to multiple processes in the workplace based on industry.

	Accident Type	Caught in Equipment or Machinery	Collision	Struck by the Object	Fire or Explosion	Crushed	Fall	Collapse	Etc.
Sources									
Equipment and machinery	Fatalities and ratio	96 (89.7%)	56 (53.3%)	50 (70.4%)	51 (96.2%)	33 (82.5%)	10 (52.6%)	5 (31.3%)	11 (73.3%)
	Construction	31	31	33	46	20	9	5	2
	Manufacturing	50	15	12	5	9	1		9
	Service	6	3	3		2			
	Etc.	9	7	2		2			
Transportation	Fatalities and ratio	6 (5.6%)	45 (42.9%)			2 (5.0%)	2 (10.5%)		
	Construction	2	29			1			
	Manufacturing	1	5						
	Service		3						
	Etc.	3	8			1	2		
Parts and materials	Fatalities and ratio	4 (3.7%)	3 (2.9%)	13 (18.3%)		3 (7.5%)		1 (6.3%)	3 (20.0%)
	Construction	2	1	11				1	1
	Manufacturing	2	1	1		3			2
	Etc.		1	1					
Structures	Fatalities and ratio	1 (0.9%)		5 (7.0%)			7 (36.8%)	10 (62.5%)	
	Construction			5			7	10	
	Manufacturing	1							
Chemicals	Fatalities and ratio				2 (3.8%)	1 (2.5%)			1 (6.7%)
	Construction				2	1			1
Products	Fatalities and ratio			2 (2.8%)					
	Construction			2					
Animals and plants	Fatalities and ratio			1 (1.4%)					
	Etc.			1					
Portable devices	Fatalities and ratio					1 (2.5%)			
	Construction					1			
Unspecified	Fatalities and ratio		1 (1.0%)						
	Construction		1						

**Table 9:** Risks and characteristics of multiple processes in the workplace based on the accident type.

	Risks and Characteristics	Risk of Fatal Accidents Caused by Multiple Processes in the Workplace Based the Industry Type	Interference Characteristics	
Accident Type			Space	Time
Caught in equipment or machinery		When working with operating (powered on) equipment or machinery, there is a risk of interference with other operations performed in the same workplace at the same time	Same workplace	Work at the same time
Collision		Risk arises when other operations are simultaneously interfered within the working radius or movement range of the equipment, machinery, or means of transport used during the process	Same workplace	Work at the same time
Struck by the object		When equipment, machinery, parts, or materials that have been displaced during work are fly-in or fall into another workspace where they are implemented at the same time	Upper and lower workplace or adjacent workplace	Work at the same time
Fire or explosion		Risk of interference when welding and cutting using operating equipment or machines at the same time in a workplace that uses inflammable substances or combustible gas	Same workplace	Work at the same time
Crushed		Risk of interference if equipment, machinery, parts, materials, or vehicles fall over during work or at the same time invade the work area being carried out in the workplace	Same workplace	Work at the same time
Fall		When equipment, machinery, parts, materials, or structures used for other processes interfere with the working space of the worker and cause a fall	Same workplace	Work at the same time or successive temporal relationships
Collapse		When a region or part, material, or structure collapses and spills out and at the same time invades the workplace	Same workplace	Work at the same time
Others		When performing other work in a space where there is lack of oxygen or that is filled with toxic gases that may cause suffocation or poisoning	Same workplace	Work at the same time or successive temporal relationships

## Data Availability

Data have been provided in the article.
